# Anisotropy and shear stress accumulation during collective migration of epithelial cells

**DOI:** 10.1007/s00249-026-01813-y

**Published:** 2026-02-02

**Authors:** Ivana Pajic-Lijakovic, Milan Milivojevic, Peter V. E. McClintock

**Affiliations:** 1 Department of Chemical EngineeringFaculty of Technology and Metallurgy, University of Belgrade, Belgrade, Serbia; 2 Department of Physics, Lancaster University, Lancaster, LA1 4YB UK

**Keywords:** Collective cell migration, Viscoelasticity, Degree of anisotropy, Strength of cell-cell adhesion contacts, Epithelial-to-mesenchymal transition

## Abstract

Anisotropy is a fundamental physical characteristic that influences efficient cell migration in biological systems. Concurrently, anisotropy serves as a primary factor contributing to a significant accumulation of mechanical stress within migrating epithelial collectives, provided that the cells maintain the strong E-cadherin-mediated cell-cell adhesion contacts that are characteristic of epithelial cells. While cells are capable of effectively enduring both compressive and tensile stress, the shear stress that can be generated during a physiological process such as collective cell migration poses a risk of: (i) disrupting the adhesion contacts among cells and between cells and the extracellular matrix, (ii) causing a partial disintegration of the lipid bilayer and cytoskeleton, (iii) triggering cellular inflammation, and (iv) inducing changes in gene expression. The principal aims of this theoretical analysis are: (i) to emphasize the main characteristics of isotropic and anisotropic wetting/de-wetting of migrating epithelial collectives as the main factor in mechanical stress generation; (ii) to formulate a constitutive model of the anisotropic viscoelasticity of migrating epithelial and mesenchymal collectives; (iii) to emphasize the physical factors related to cell sensitivity to shear stress; and (iv) to explore potential cellular strategies to mitigate shear stress, while also highlighting the associated costs of these strategies.

## Introduction

The process of tissue rearrangement during morphogenesis, wound healing, and cancer invasion is contingent upon the synchronized migration of cellular clusters (Clark and Vignjevic, 2015; Barriga et al 2018; Barriga and Mayor 2019; Pajic-Lijakovic and Milivojevic 2021a). The effectiveness with which these clusters can reach their designated tissues is dependent on their capacity for coordinated and cooperative migration (Barriga and Mayor 2019; Shellard and Mayor 2019). Coordination among cells is associated with directional movement, while the cooperation of clusters is influenced by the nature of cell-cell adhesion interactions. Directional movement is typically guided by a range of external signals, including chemical, mechanical, or electrical cues, which direct cells on their migratory paths (Murray et al. 1988; Shellard and Mayor, 2019). Consequently, an established gradient of: (1) soluble chemical cues induce chemotaxis, (2) an electric field induces galvanotaxis, (3) the stiffness of the substrate matrix or adjacent tissue induces durotaxis, and (4) cellular adhesion sites or substrate-bound cytokines induce haptotaxis (Murray et al. 1988; Shellard and Mayor, 2019). Cell signaling also contributes to directional cell migration (Petrungaro et al. 2019). Directional migration of polarised cells can be characterized by their degree of anisotropy (i.e., the fraction of cells aligned in the direction of migration), which is inhomogeneously distributed along epithelial monolayers. ’Anisotropy refers to a characteristic of a system in which its physical properties differ with the direction of measurement. This indicates that a system may display varying attributes, such as strength or stiffness, along different axes (**Glossary**). Higher degrees of anisotropy — in traction forces, cell polarity, velocity, mechanical stress or substrate alignment — promote force transmission over long distances (Serra-Picamal et al. 2012). This enables tissue-scale coordination, where cells far from each other can still migrate coherently. Intercellular force propagation in epithelial monolayers is highly dependent on the anisotropy of F-actin organization and the force distribution across cell-cell adhesion contacts (Ruppel et al. 2023). The anisotropic nature of the force distribution across cell-cell adhesion complexes is regulated by α-catenin, which promotes actin polymerization (Matsuzawa et al. 2018). Anisotropic cytoskeletal arrangements enable efficient force transmission across cells (Fang et al. 1993). Epithelial cells can utilize their size, shape and internal structure through contractile forces produced by stress fibers to modulate their response to mechanical stress and associated biological activities (López-Gay et al. 2020; Fang et al. 2023). Although intercellular adhesion and the chemo-mechanical feedback associated with actomyosin contraction are recognized as regulators of cell shape, recent findings underscore the importance of cell-substrate interactions. In particular, the traction forces produced by F-actin networks play a vital role in orchestrating collective motion (Xu et al. 2005). These contractile forces influence cell tractions which have a feedback impact on cell rearrangement via collective cell migration (Saraswathibhatla and Notbohm, 2020). Cell rearrangement caused by mechanical stress generation is influenced by cell signaling. Morphogens (i.e., signaling molecules) provide positional information during tissue rearrangement. (Stapornwongkul et al. 2020) highlighted that synthetic morphogens have the potential to be utilized for programming *de novo* multidomain tissue patterns.

An inhomogeneous distribution of the degree of anisotropy is caused primarily by the accumulation of cell mechanical stress (Serra-Picamal et al. 2012; Notbohm et al. 2016; Bazellières et al. 2015). Regions with a reduced degree of anisotropy correlate with regions that display increased cell packing density, which is marked by significant cell-cell interactions. Conversely, for the process of ordered (anisotropic) cell migration, the cell packing density must be less than or equal to that observed in a confluent state. Cells tend to migrate from regions of low anisotropy (more isotropic, disordered) to high anisotropy (more aligned) regions by following the gradient of cell packing density (Lin et al. 2025).

Collective cell migration induces the generation of mechanical stress (**Glossary**), which has a feedback impact on cell migration itself (Serra-Picammal et al. 2012; Saw et al. 2017; Pajic-Lijakovic et al. 2024). The cell jamming state transition (i.e., cell contractile to non-contractile state transition), live cell extrusion, and epithelial-to-mesenchymal transitions are all possible outcomes induced by the accumulation of mechanical stress during collective cell migration (Serra-Picamal et al. 2012; Saw et al. 2017; Pajic-Lijakovic et al. 2024). The central question is whether epithelial cells respond to stress by transitioning from anisotropic to isotropic migration while retaining their epithelial phenotype—ultimately leading to the jamming state under increased compressive stress—or whether they instead undergo an epithelial-to-mesenchymal transition that allows them to maintain anisotropic migration to some extent. All of these outcomes influence energy storage and dissipation within epithelial monolayers in different ways, which have a feedback impact on their viscoelasticity and alignment. These outcomes are related to cell strategies for decreasing an undesirable accumulation of mechanical stress. The main focus of this review is to analyse the physical aspects of this complex phenomenon. The migration of epithelial monolayers induces the generation of normal stress components (tensional or compressive) (**Glossary**) up to a few hundreds of Pa, while the generated shear stress is up to several tens of Pa (Serra-Picamal et al. 2012; Tambe et al. 2013). Cells tend to elongate and polarize along the tensile direction by following the direction of maximum principal stress (Trepat and Fredberg 2011). Consequently, cell migration occurs along the tension gradient. The phenomenon is known as plithotaxis (Trepat and Fredberg, 2011). Shear stress (**Glossary**) can cause cell reorientation via torques exerted on the cytoskeleton and adhesion junctions (Saw et al. 2017; Pajic-Lijakovic et al. 2024). Cells may polarise perpendicularly to the compressive axis to escape compression (Blanchard et al. 2024). An increase in compressive stress accompanied by an increase in cell packing density can lead to the jamming state transition (Pajic-Lijakovic et al. 2024). An increase in cell packing density intensifies cell-cell head-on interactions and consequently, contact inhibition of locomotion causing weakening of cell-cell and cell-matrix adhesion contacts (Roycraft and Mayor 2016). Cell jamming takes place when the interval between two direct interactions is less than the time required for cell re-polarisation (Pajic-Lijakovic and Milivojevic 2021b). Under these circumstances, cells undergo a transition from a contractile to a non-contractile state by enhancing the strength of focal adhesions (Gupton et al. 2006). As a result, cell-cell interactions: (i) lead to a decrease in the degree of anisotropy and (ii) induce more energy dissipation. Cell compressive stress accompanied by cell shear stress can induce live cell extrusion (Saw et al. 2017; Pajic-Lijakovic et al. 2024) The dissipation of energy, caused by remodeling of cell-cell and cell-matrix adhesion contacts, results in a decrease in mechanical stress (Pajic-Lijakovic and Milivojevic 2025).

Cells can withstand compressive stress levels of several kPa; however, exposure to shear stress as low as a few Pa may lead to: (i) a reduction in cytoskeletal stiffness (Flitney et al. 2009), (ii) activation of pro-inflammatory pathways (Pitenis et al. 2018), (iii) modifications in gene expression (Espina et al. 2023), and (iv) impairment of intercellular junctions (Maggiorani et al. 2015) and focal adhesions between epithelial cells and the extracellular matrix (Saw et al. 2017), along with the disruption of the lipid bilayer (Yamamoto and Ando 2015).

Shear stress can induce the epithelial-mesenchymal transition (EMT), a process where epithelial cells lose their apical-basal polarity, and adhesion properties, gaining migratory and invasive capabilities (Lamouille et al. 2014; Gandalovičová et al. 2016; Yang et al. 2020). The EMT is associated with the weakening of cell-cell adhesion contacts, reorganization of the cytoskeleton, and a shift in the signalling pathways that regulate cell morphology and gene activity, ultimately leading to enhanced cell motility and acquisition of the mesenchymal phenotype (Lamouille et al. 2014). Cells exhibiting different levels of mesenchymal characteristics, including variations in cell polarity, motility, and the strength of cell-cell adhesion, may develop as a consequence of a partial EMT (Barriga and Mayor 2019; Yang et al. 2020).

Although the generation of mechanical stress during collective cell migration has been extensively studied, the influence of anisotropy on this mechanical stress generation is only beginning to be understood. While directional, anisotropic cell movement is more efficient than random isotropic movement, it results in more intensive generation of cell mechanical stress (Bazellières et al. 2015). Given the susceptibility of epithelial cells to shear stress, it is important to identify the ways in which cells minimise the generation of shear stress during collective cell migration. The main goal of this theoretical consideration is to point out: (i) the relationship between the degree of anisotropy and the generation of additional shear stress, (ii) the impact of the generated cell compressive stress and increased cell packing density on the extent of the anisotropy, (iii) the impact on cell rearrangement of an inhomogeneous distribution of the degree of anisotropy, and (iv) possible ways in which cells reduce the shear stress. As the first step, it is necessary to point out how mechanical stress is accumulated during collective cell migration and then to indicate possible physical factors that influence the sensitivity of epithelial cells to shear stress.

## Mechanical stress generation during collective cell migration

Collective cell migration induces generation of mechanical stress. The generation of mechanical stress is more intense in anisotropic parts of monolayers than in isotropic ones (Bazellières et al. 2015). While only the $\:{\epsilon\:}_{xx}$ component contribute to the generation of the normal stress component in the *x*-direction $\:{\sigma\:}_{xx}$ in isotropic parts, all strain components $\:{\epsilon\:}_{xx}$, $\:{\epsilon\:}_{yy}$, and $\:{\epsilon\:}_{xy}$ contribute to the generation this stress component $\:{\sigma\:}_{xx}$ in anisotropic parts of monolayers. Similar relationships can be postulated for other stress components in the case of anisotropic parts by including all strain components.

Before formulating the constitutive stress-strain model, it is necessary to discuss scenarios for generation of mechanical stress caused by epithelial wetting/de-wetting.

### Epithelial wetting/de-wetting during collective cell migration

Epithelial monolayers, like other soft matter systems, undergo wetting (extension) or de-wetting (compression) on substrate matrices, depending on the interrelationship between the cohesion properties of the system and matrix as quantified by their surface tensions, as well as the adhesion between them, expressed in the form of the spreading factor (de Gennes 1985). The latter depends on the surface tensions of the system and matrix, as well as on the interfacial tension between them. In contrast to other soft matter systems, multicellular systems actively change their surface tension and cell-matrix interfacial tension by actomyosin contractions and by the remodelling of cell-cell and cell-matrix adhesion contacts during collective cell migration (Pérez-González et al. 2019). However, passive mechanical processes such as Poisson’s effect also contribute to the wetting/de-wetting of epithelial monolayers, depending on the degree of anisotropy (Pajic-Lijakovic et al. 2025b).

**Fig. 1:**
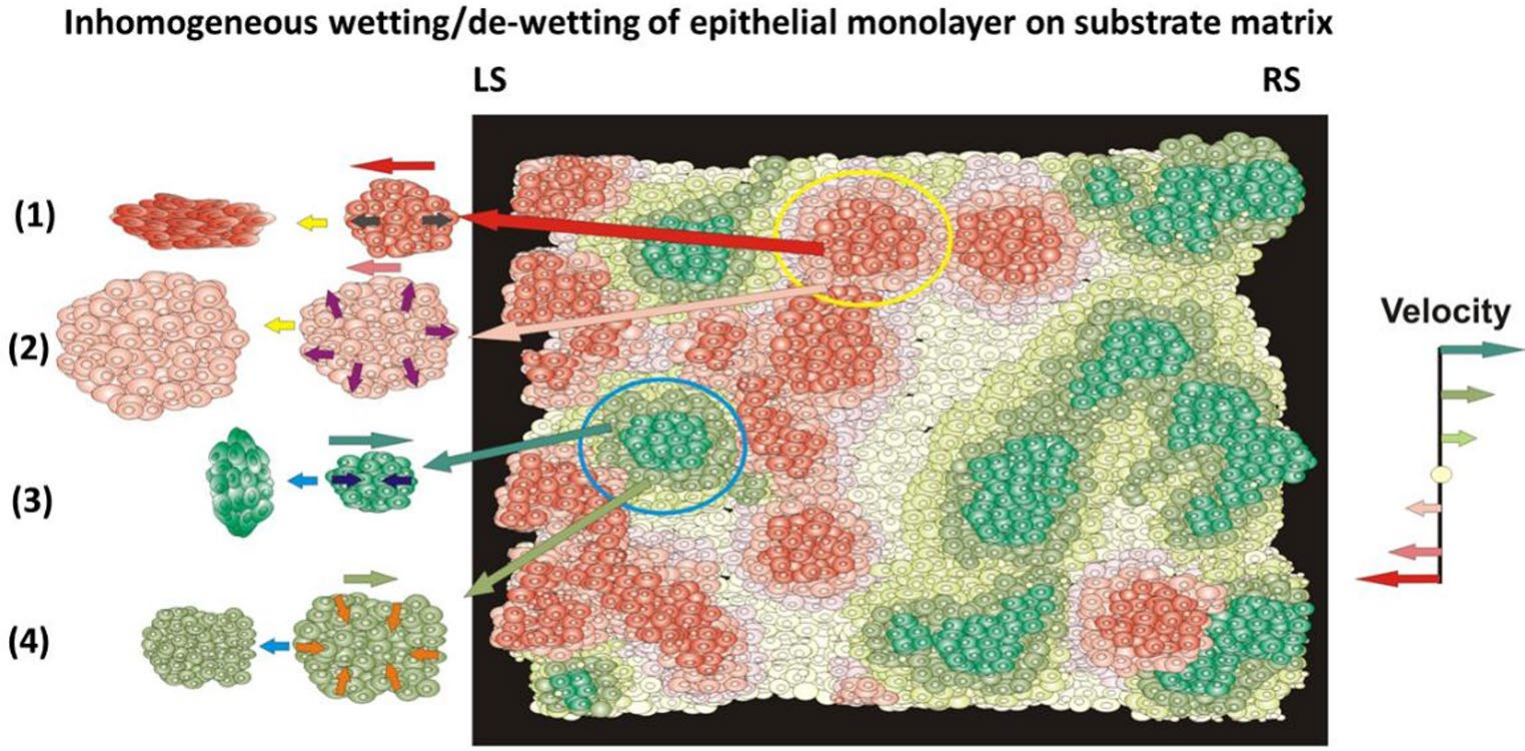
Schematic representation of wetting/de-wetting of an epithelial monolayer on a substrate matrix inspired by the experimental data of Serra-Picamal et al. (2012). Green domains undergo translation towards left on the left side of the monolayer (LS) moving at various velocities labelled by green arrows. Red domains undergo translation towards right motion on the right side of the monolayer (RS) moving at various velocities labelled by red arrows. Some red domains undergo translation backwards on their RS, while some green domains undergo translation backwards on their LS. Dark green/red domains are anisotropic and satisfy the condition that the cell packing density is $\:{n}_{e}\le\:{n}_{conf}$, while light green/red domains are isotropic and satisfy the condition that the cell packing density is $\:{n}_{e}>{n}_{conf}$. Isotropic domains move slower than anisotropic domains. Grey domains are in the state near jamming with the cell packing density $\:{n}_{e}\to\:{n}_{j}$ (where $\:{n}_{j}\gg\:{n}_{conf}$). Besides translation forwards/backwards anisotropic domains perform uni-axial extension/compression, while isotropic domains perform biaxial extension/compression such that $\:{\epsilon\:}_{xx}={\epsilon\:}_{yy}$ (where $\:{\epsilon\:}_{xx}$ and $\:{\epsilon\:}_{yy}$ are the normal strain components). Domains on the LS of the monolayer undergo: (**1**) uni-axial extension, (**2**) biaxial extension, (**3**) uni-axial compression, and (**4**) biaxial compression. While red domains on the LS of the monolayer undergo extension, red domains on the RS of the monolayer undergo compression. While green domains on the LS undergo compression, green domains on the RS undergo extension. Red domains have net motion to the left, and green domains net motion to the right, at velocities indicated by the colour key on the right-hand side of the main diagram; superimposed on these net velocities are local relative velocities that change the shape of each domain corresponding to the wetting or de-wetting processes. (Yellow and blue horizontal arrows represent the passage of the time during the deformation process of the domains, while dark and light red and green arrows point out the direction of translator movement of the domains)

**Fig. 2:**
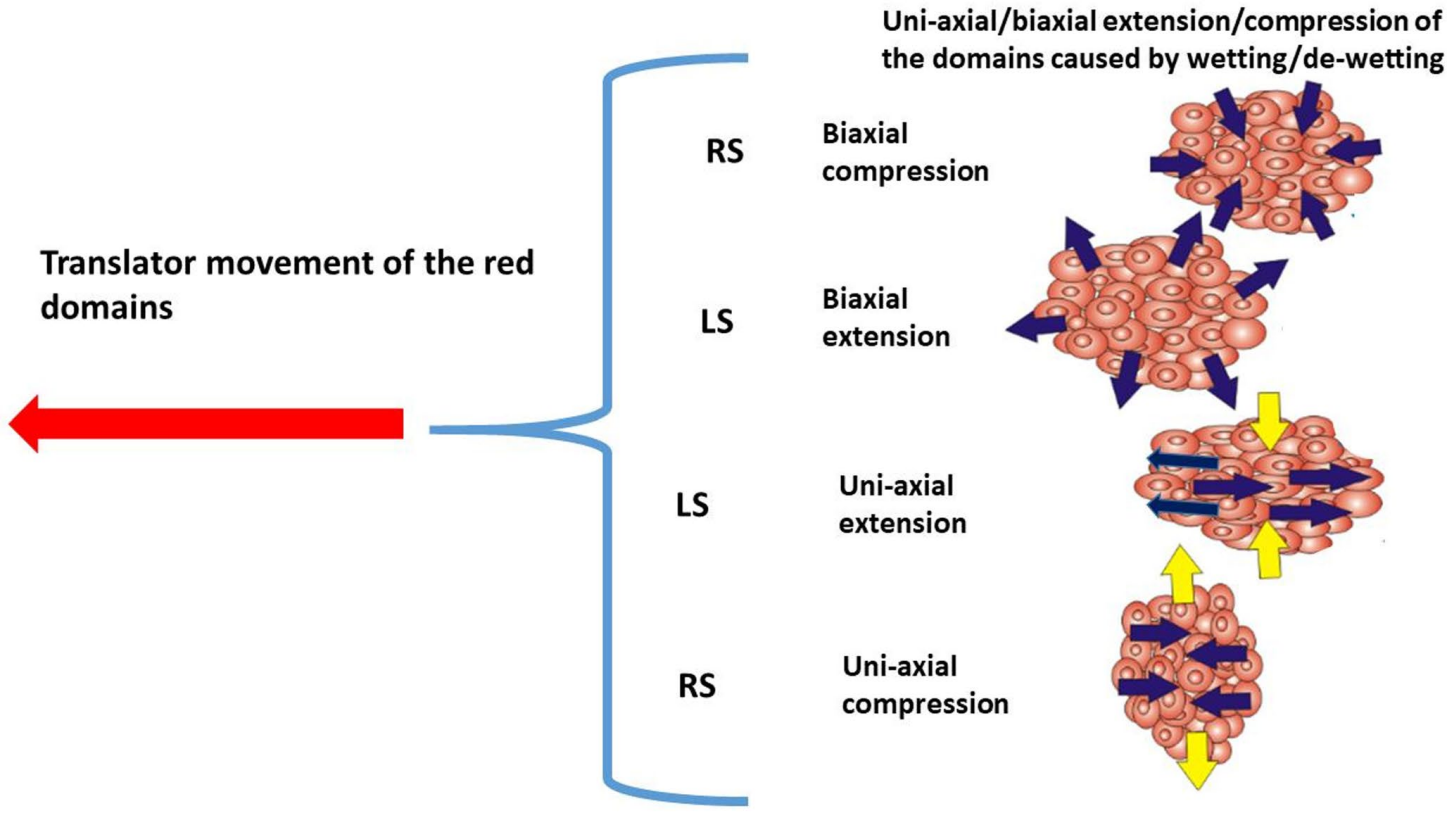
Movement of cells within the multicellular domains: (i) active translation via collective cell migration, and (ii) extension/compression of the domains via collective cell migration, which can be uni-axial for anisotropic domains and biaxial for isotropic domains which satisfies the condition that $\:{\epsilon\:}_{xx}={\epsilon\:}_{yy}$ (where $\:{\epsilon\:}_{xx}$ and $\:{\epsilon\:}_{yy}$ are the normal strain components). (Dark blue arrows mark the active wetting/de-wetting of domains caused by collective cell migration, while yellow arrows mark passive wetting/de-wetting of anisotropic domains in the direction perpendicular to cell migration caused by Poisson’s effect)

The main characteristics of epithelial monolayers are inhomogeneous distributions of: (i) the strengths of cell-cell and cell-matrix adhesion contacts, (ii) cell tractions, (iii) cell contractility, (iv) cell packing density, (v) cell velocity and (vi) mechanical stress, leading to inhomogeneous distribution of epithelial surface tension and epithelial-matrix interfacial tension thereby causing inhomogeneous wetting/de-wetting (Serra-Picamal et al. 2012; Tlili et al. 2018; Pérez-González et al. 2019; Pajic-Lijakovic et al. 2025a). The inhomogeneous wetting/de-wetting of epithelial monolayers is shown schematically in Fig. 1:

Epithelial fingering is an example of inhomogeneous wetting/de-wetting of epithelial monolayers (Blanch-Mercader et al. 2017; Alert et al. 2019; Trenado et al. 2021). Intensive epithelial wetting correlates with the stronger cell tractions that are pronounced at the edges of monolayers. We build on these experimental and theoretical insights by incorporating the viscoelastic properties of epithelial monolayers for both anisotropic and isotropic conditions. Consequently, we treat epithelial monolayers as ensembles of multicellular domains, each characterized by an approximately homogeneous distribution of these physical parameters (**Glossary**). Such domains are unstable. They exist for some period of time and then lose their identity due to interactions among domains. Consequently, wetting and de-wetting behavior may vary from one domain to another. Some domains undergo wetting, while the others at the same time undergo de-wetting and generate local forward and backward flows (Serra-Picamal et al. 2012). Each domain has two degrees of freedom: (i) spatial deformation and (ii) translational displacement of the center of mass of the domains as shown in Fig. 2:

The deformation can be uni-axial or biaxial, depending on the degree of anisotropy. It induces the generation of mechanical stress depending on the domain viscoelasticity.

Whether a given domain undergoes wetting or de-wetting can be described by the magnitude of the epithelial spreading factor. The spreading factor of epithelial monolayers is formulated in **Box 1.**

**Box 1.** Spreading factor of epithelial monolayers. The phenomena of epithelial wetting and de-wetting are influenced by the interplay of specific adhesion and cohesion energies, which can be quantified by the spreading factor: $\:{S}_{e}={e}_{a}-{e}_{c}$ (where $\:{e}_{a}$ is the epithelial-matrix adhesion energy and $\:{e}_{c}$ is the cohesion energy) (Pajic-Lijakovic et al. 2025a). The cell-matrix adhesion energy depends on the strength of the cell-matrix adhesion contacts and has been expressed as: $\:{e}_{a}\left(r,\tau\:\right)={\rho\:}_{a}\frac{1}{2}{k}_{FA}{\left|{\overrightarrow{\boldsymbol{u}}}_{\boldsymbol{m}}\right|}^{2}$ where $\:{\rho\:}_{a}$ is the surface density of cell-substrate adhesion contacts, $\:{k}_{FA}$ is the elastic constant of single cell-matrix focal adhesion, and $\:{\overrightarrow{\boldsymbol{u}}}_{\boldsymbol{m}}$ is the matrix displacement field (Murray et al. 1988). The cohesion energy represents the work needed to separate two connected epithelial surfaces and depends on the strength of cell-cell adhesion contacts and cell contractility. This energy per unit surface is correlated with the epithelial surface tension, i.e. $\:{e}_{c}=2{\gamma\:}_{e}$ (Pajic-Lijakovic et al. 2025a). Given that the relationship between dynamic epithelial surface tension and variations in epithelial surface area can be examined through the lens of dilation viscoelasticity (Babak et al. 2005; Pajic-Lijakovic et al. 2023a), it is important to note that the spreading factor is indicative of the viscoelastic properties of epithelial monolayers. The epithelial surface tension also depends on the deformation of the multicellular surfaces. While stretching results in an increase in the epithelial surface tension, compression causes a weakening of cell-cell adhesion contacts and, consequently, a decrease in the epithelial surface tension (Guevorkian et al. 2021; Pajic-Lijakovic et al. 2025a). When the spreading factor $\:{S}_{e}>0$, cells undergo wetting. Otherwise, for $\:{S}_{e}<0$, cells undergo de-wetting (Pajic-Lijakovic et al. 2025a). The spreading factor is inhomogeneously distributed along the monolayers caused primarily by the inhomogeneous distribution of the strength of cell-cell and cell-matrix adhesion contacts and strain (Serra-Picamal et al. 2012; Pérez-González et al. 2019). Epithelial wetting/de-wetting is an oscillatory phenomenon (Serra-Picamal et al. 2012; Pajic-Lijakovic et al. 2024) that can be described in a sequence of inter-connected steps:

• Initially, at $\:\tau\:=0$ the spreading factor of the epithelial monolayer satisfies the conditions: (i) $\:{S}_{e}\left(r,\tau\:\right)>0$ (where $\:r=r\left(x,y\right)$ is the space coordinate of the monolayer such that $\:r\le\:R\left(\tau\:\right)$ and $\:R\left(\tau\:\right)$ is the coordinate of the monolayer edge) and (ii) $\:{S}_{e}\left(R,\tau\:\right)\to\:max$ caused by intensive tractions of cells near the edge. Consequently, epithelial cells undergo wetting, which is pronounced near the monolayer edge (Serra-Picamal et al. 2012).

• Wetting-induced stretching of the monolayer, being more intensive near the edge, causes an increase in the epithelial surface tension and in the cohesion energy relative to the adhesion energy. This increase in $\:{e}_{c}$ results in a decrease in the spreading factor along the edge of the monolayer.

• When the spreading factor of the domains near the edge becomes $\:{S}_{e}\left(R,\tau\:\right)<0$, these domains undergo de-wetting, while the domains located in the central regions have satisfied the condition that $\:{S}_{e}\left(r,\tau\:\right)>0$ and undergo wetting. This state of the monolayer can be characterised as partial de-wetting (Serra-Picamal et al. 2012; Pajic-Lijakovic et al. 2024).

• Collision of forward flow, caused by wetting, and backward flow, caused by de-wetting, induces: (i) an increase in compressive stress accompanied by the cell packing density leading to a weakening of cell-cell adhesion contacts caused by contact inhibition of locomotion (Roycraft and Mayor 2016) and (ii) a decrease in the degree of anisotropy. This collision of opposite cell fronts can lead to the cell-jamming state transition or to live cell extrusion (Saw et al. 2017; Pérez-González et al. 2019; Pajic-Lijakovic et al. 2024).

During the processes of epithelial wetting and de-wetting, the domains of the monolayer can undergo either uni-axial or biaxial extension or compression (Fig. 1). Biaxial extension and compression are indicative of isotropic domains, whereas uni-axial extension and compression are characteristic of anisotropic domains. In instances of biaxial extension and compression resulting from collective cell migration, the wetting and de-wetting processes are considered to be active. Conversely, in the case of uni-axial extension and compression, the wetting and de-wetting process encompasses both active and passive contributions. The active process occurs in the direction of cell migration, while the passive process occurs in a direction that is perpendicular to that of migration. This passive process is associated with the effects described by Poisson (Pajic-Lijakovic et al. 2024). The main characteristics of wetting and de-wetting of isotropic and anisotropic domains depending on the Poisson’s ratio (**Glossary**) and degree of anisotropy are discussed in **Box 2**.

**Box 2**.Wetting/de-wetting of isotropic and anisotropic epithelial domains. Wetting/de-wetting of the domains causes the generation of uni-axial/bi-axial extensional/compressional strain depending on the degree of anisotropy $\:\alpha\:$ (Pajic-Lijakovic et al., 2024). Boundary conditions can be established for: (i) the isotropic case $\:\alpha\:=0$ and (ii) the anisotropic case $\:\alpha\:=1$. Extension/compression of isotropic domains is equal in all directions and can be treated as biaxial. However, extension/compression of anisotropic domains with the degree of anisotropy $\:\alpha\:=1$ is rather uniaxial. Consequently, two scenarios can be considered:

(a) In isotropic domains where $\:\alpha\:=0$, the normal strain component in the *x*-direction, denoted as $\:{\epsilon\:}_{xx}$, is equivalent to the normal strain component in the *y*-direction, represented as $\:{\epsilon\:}_{yy}$, such that $\:{\epsilon\:}_{yy}={\epsilon\:}_{xx}$. These strain components can be categorized as: (i) extensional, where $\:{\epsilon\:}_{yy}>0$ and $\:{\epsilon\:}_{xx}>0$, indicating an active wetting condition, or (ii) compressive, where $\:{\epsilon\:}_{yy}<0$ and $\:{\epsilon\:}_{xx}<0$, indicating a de-wetting condition.

(b) In the context of anisotropic domains ($\:\alpha\:=1$), it is observed that when cells migrate along the *x*-direction, the relationship $\:{\epsilon\:}_{yy}=-\nu\:{\epsilon\:}_{xx}$ holds, where *ν* represents the viscoelastic, space-time-dependent Poisson’s ratio (Pajic-Lijakovic et al. 2025b). The average Piosson’s ratio of Madin-Darby Canine Kidney-MDCK and HeLa epithelial monolayers is $\:\nu\:\sim0.77$ (Moisdon et al. 2022). When the Poisson’s ratio $\:\nu\:>0.5$, extension in the *x*-direction induces compression in *y*-direction and vice versa. It means that: (i) wetting can be characterised by $\:{\epsilon\:}_{xx}>0$ and $\:{\epsilon\:}_{yy}<0$ and (ii) de-wetting can be characterised by $\:{\epsilon\:}_{xx}<0$ and $\:{\epsilon\:}_{yy}>0$. While cells perform active wetting/de-wetting in the direction of cell migration, passive wetting/de-wetting occurs in the perpendicular direction.

Both scenarios can be incorporated in terms of the following relationship between two normal strain components, expressed as:

1 $$\:{\epsilon\:}_{yy}\left(\alpha\:,\nu\:\right)=\left(1-\alpha\:-\alpha\:\nu\:\right){\epsilon\:}_{xx}$$

Besides normal strain components, a shear strain component $\:{\epsilon\:}_{xy}$ is generated within the domains and along the border between domains. All strain components contribute to the generation of every component of mechanical stress depending on the degree of anisotropy.

Consequently, wetting/de-wetting of epithelial domains depends on the degree of anisotropy. While the shape of anisotropic domains is elliptical, the shape of isotropic domains is more circular. It is in accordance with fact that wetting/de-wetting is more intensive in the direction of cell migration than in the perpendicular direction of anisotropic domains. The epithelial spreading factor as a function of the degree of anisotropy can be expressed by the modified equation proposed by (Pajic-Lijakovic et al. 2025a) as:

2 $$\:{S}_{e}\left(\alpha\:,\theta\:\right)={S}_{e}^{0}+\alpha\:\left(\varDelta\:{e}_{a}\left(\theta\:\right)-\varDelta\:{e}_{c}\left(\theta\:\right)\right)$$

where $\:{S}_{e}^{0}$ is the spreading factor for isotropic cell movement, $\:\alpha\:$ is the degree of anisotropy, $\:\varDelta\:{e}_{a}\left(\theta\:\right)$ and $\:\varDelta\:{e}_{c}\left(\theta\:\right)$ are anisotropic contributions to the adhesion and cohesion energies, respectively such that $\:\left|\varDelta\:{e}_{a}\left(\theta\:\right)-\varDelta\:{e}_{c}\left(\theta\:\right)\right|\to\:max$ for $\:\theta\:={\theta\:}_{0}$, $\:\mathrm{a}\mathrm{n}\mathrm{d}\:{\theta\:}_{0}$ is the principal axis of anisotropy, i.e., the direction of cell migration. The degree of anisotropy strongly affects the generation of strain during epithelial wetting/de-wetting. It is necessary to emphasise the impact of the degree of anisotropy to generated strain before formulating an appropriate stress-strain constitutive model.

### Scenarios of mechanical stress generation

**Fig. 3:**
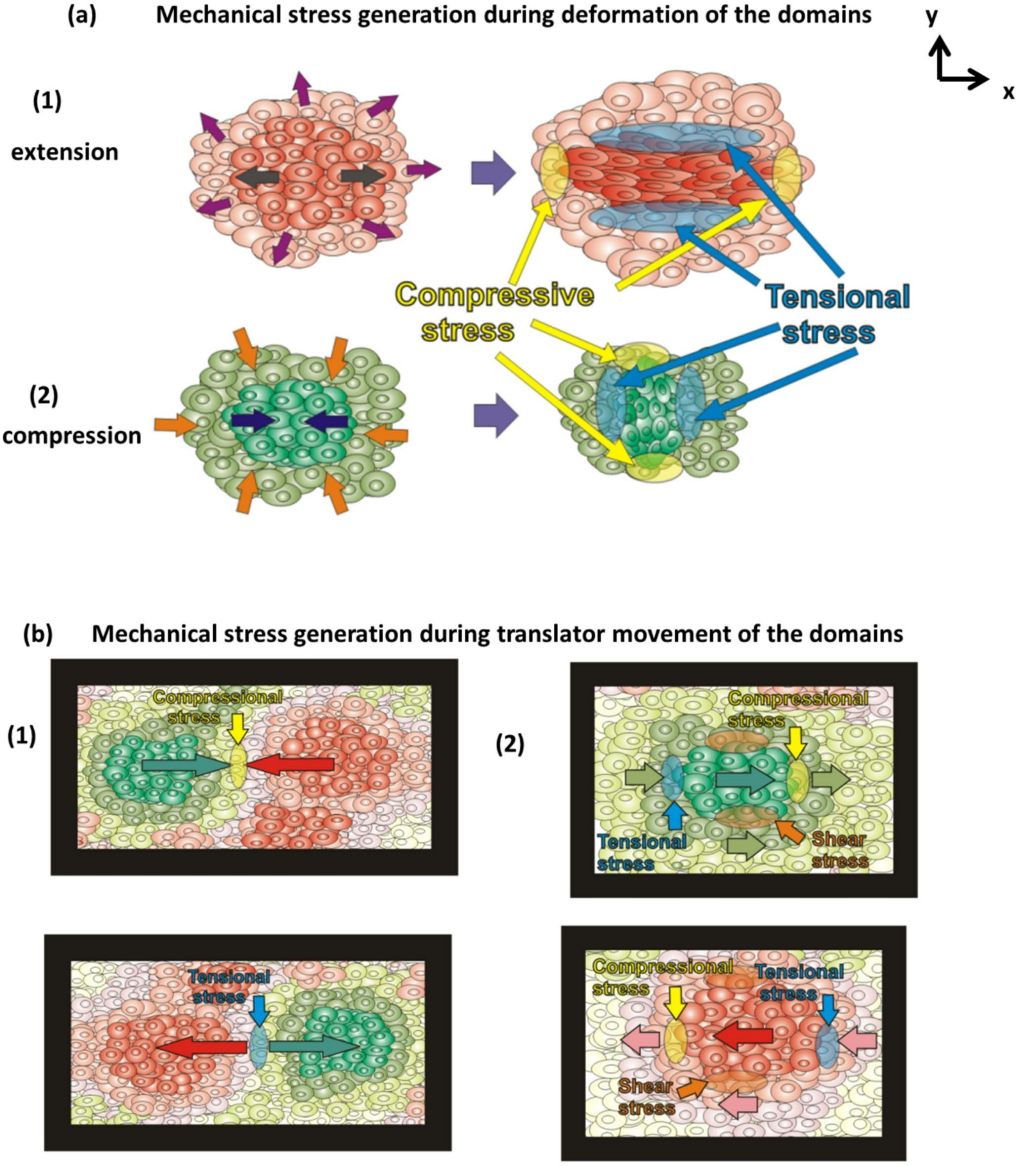
Schematic presentation of interactions among multicellular domains during epithelial wetting/de-wetting, which lead to generation of mechanical stress:

Inhomogeneous wetting/de-wetting causes generation of mechanical stress within migrating epithelial monolayers. The scenarios of accumulation pf mechanical stress as a product of interactions among multicellular domains are shown in Fig. 3:

1. Mechanical stress generation caused by extension/compression of the domains: (1) **Extension of isotropic and anisotropic domains**: More intensive extension of anisotropic domains relative to isotropic domain near the monolayer edge can induce compression of the domains behind them along the border in the *x*-direction leading to generation of compressive stress. Compression of anisotropic domain caused by Poisson’s effect and extension of isotropic domain, in the y-direction, lead to generation of tensional stress; (2) **Compression of isotropic and anisotropic domains**: More intense compression of an anisotropic domain relative to the isotropic domain near the monolayer edge can induce extension of the domains behind them along the border in the x-direction leading to generation of tension stress. Extension of anisotropic domain caused by Poisson’s effect and compression of isotropic domain, in y-direction, lead to generation of compressive stress. (Violet arrows mark biaxial extension of isotropic domains; black arrows mark uni-axial extension of anisotropic domains; orange arrows mark biaxial compression of isotropic domains; dark blue arrows mark uni-axial compression of anisotropic domains).
2. Mechanical stress can be generated by translation of the domains: (1) collision of forward- and backward-migrating domains generates compressive stress, while movement of the domains in opposite directions generates tensional stress, and (2) movement of the domains with various speeds leads to the generation of all stress components. (Red and green arrows mark the directions of translation of the domains, while yellow, blue and orange arrows mark the contact zones in which stress is generated).

- Tensional stress is generated in the *x*-direction during epithelial wetting (i.e., in the direction of cell migration) within anisotropic domains, and in all directions within isotropic domains. Tensional stress may arise in the *y*-direction, perpendicular to the cell migration direction, during the de-wetting process with anisotropic domains aligned in the *x*-direction (Fig. 3a). Additionally, it can occur in the *y*-direction at the border between two anisotropic domains, as well as at the border between isotropic and anisotropic domains during the wetting process. It is in accordance with the fact that anisotropic domains undergo uniaxial extension in the *x*-direction and compression in the *y*-direction caused by Poisson’s effect (Pajic-Lijakovic et al. 2025b). Compression of neighbouring anisotropic and isotropic domains in the *y*-direction toward the centres of these domains generates tensional stress within the border between these domains. Tensional stress can be generated at the contact point between neighbouring domains during their migration in opposite directions (Fig. 3b). Movement of surrounding domains with various speeds result in the generation of all stress components.
- Compressive stress is generated in the *x*-direction during epithelial de-wetting within anisotropic domains, and in all directions within isotropic domains. Intensive wetting of anisotropic domains near the monolayer edge can induce compression of the domains behind them along the border in the *x*-direction (Fig. 3a). Inhomogeneous wetting of isotropic domains can generate compressive stress along the border between neighbouring domains. Compressive stress can be generated during collision between the domains that migrate in the opposite directions (Fig. 3b).
- Shear stress is generated more intensively in anisotropic domains compared to isotropic ones during the processes of wetting and de-wetting. It is in accordance with fact that compressive and extensional strain components, accompanied by shear strain, contribute to the generation of shear stress. This stress component is also generated along the border between neighbouring domains that tend to move at different speeds: either in the same direction or in opposite direction (Fig. 3b).

To articulate the relationship between stress components and strain components, as well as the degree of anisotropy, it is necessary to establish an appropriate constitutive model by pointing out the main features of the viscoelasticity caused by collective cell migration.

### Cell hypersensitivity to shear stress: the causes

Cells can withstand compressive stress levels of several kPa; however, shear stress at just a few Pa may disrupt cell structure. Lower levels of shear stress are more likely to activate Rac1 and Cdc42, which in turn promote cell polarity and the formation of lamellipodia (Wojciak-Stothard and Ridley, 2003). While shear stress promotes cell polarity, compressive stress supresses the cell polarity that arises from cell-cell interactions (Pajic-Lijakovic et al. 2024). The cellular response to shear stress is influenced by both the intensity of the stress and the rate at which it changes (Bilek et al. 2003). Physiological processes such as a collective cell migration generate shear stress of a few tens of Pa. (Tambe et al. 2013) quantified the maximum shear stress produced during the collective movement of MDCK cell monolayers on substrate matrices with a magnitude of 100 Pa. However, shear stress of a few tens of Pa can induce inflammation of epithelial cells and even cell death (Pitenis et al. 2018; Pitenis and Sayer 2020).

The apical membrane of epithelial cells is relatively soft and unprotected, especially in non-specialized epithelia (unlike endothelium, which adapts better to shear). Higher shear stress can induce: (i) membrane shear thinning and rupture (Yamamoto and Ando, 2015), (ii) internalisation of membrane proteins such as e.g., E-cadherin, which has a feedback impact on the cell signalling pathway (Lawler et al. 2009; He et al. 2018), (iii) loss of cilia and microvilli (Maggiorani et al. 2015), (iv) disruption of cell-cell adhesion contacts (Maggiorani et al. 2015), (v) a softening of the cytoskeleton (Flitney et al. 2009), and (vi) perturbation in gene expression (Espina et al. 2023). While uniaxial stretching and hypotonic swelling decreases the fluidity of the lipid bilayer, shear stress decreases the membrane lipid order and increases membrane fluidity (Yamamoto and Ando, 2015). Internalisation of the membrane proteins also has a feedback impact on the rearrangement of the cytoskeleton, as well as membrane viscoelasticity (He et al. 2018). Consequently, shear stress mainly affects the fluidity and order of the lipid bilayer, while compression induces buckling or wrinkling of the bilayer by reducing the mobility of lipids leading to an increase in bending rigidity and the lipid bilayer’s resistance to mechanical loading (Purushothaman et al. 2015).

Shear stress disorders the state of the cytoskeletal filaments. Some of them are stretched while their neighbours are simultaneously compressed. The response of single filaments depends on their current orientation relative to the direction of the external shear stress. Stretched semi-flexible filaments exert higher forces than compressed ones under the same absolute deformation (Broedersz and MacKintosh 2014). The phenomenon is accompanied by a nonlinear force change during the stretching of semi-flexible filaments caused by enthalpic effects, and an almost linear force change during compression of single semi-flexible filaments caused by entropic effects. The behaviour of semi-flexible filaments under stretching/compression was described by a worm-like chain model (Yamakawa 1971). Altered structural changes of filaments cause an inhomogeneous accumulation of strain energy, which can induce softening and a partial disintegration of the cytoskeleton (Espina et al. 2023). In contrast to shear stress, compressive stress induces more homogeneous energy storage within the cytoskeleton and a lower strain energy per single filament.

Given that cells exhibit extreme sensitivity to shear stress, it is essential to explore how the anisotropic nature of cell migration contributes to its generation, as well as to try to identify the strategies employed by cells to mitigate the effects of shear stress.

## Stress-strain constitutive model depending on the degree of anisotropy

Collective cell migration results in energy storage and dissipation, characterized by viscoelasticity (Pajic-Lijakovic and Milivojevic 2021b). The mechanism of energy dissipation has a feedback impact on cell rearrangement. Intensive energy dissipation has the potential to decrease the undesirable accumulation of mechanical stress. Energy dissipation can be controlled by the remodeling of cell-cell and cell-matrix adhesion contacts (Pajic-Lijakovic et al. 2024).

Migrating epithelial collectives have been treated as viscoelastic solids, primarily through the establishment of strong E-cadherin-mediated cell-cell adhesion contacts (Pajic-Lijakovic et al. 2024). Development of the corresponding viscoelasticity for a cell packing density $\:{n}_{e},$ equal to or lower than the cell packing density for cells in the confluent state $\:{n}_{conf}$, is a multi-time process. Petitjean et al. (2010) demonstrated that the MDCK cell monolayers attained confluence at a cell packing density of approximately $\:{n}_{conf}\sim2.5x{10}^{5}\:\frac{\mathrm{c}\mathrm{e}\mathrm{l}\mathrm{l}\mathrm{s}}{{\mathrm{c}\mathrm{m}}^{2}}$ and a cell velocity of around $\:\sim0.14\:\frac{{\upmu\:}\mathrm{m}}{\mathrm{m}\mathrm{i}\mathrm{n}}$. The cell velocity, induced strain, and residual stress accumulation change over hours, while cell stress relaxation occurs over minutes (Marmottant et al. 2009; Khalilgharibi et al. 2019; Pajic-Lijakovic et al. 2024). It means that the stress increases and relaxes toward the residual stress over minutes in the form of successive short-time relaxation cycles under constant strain per cycle, while the strain change is much slower (Pajic-Lijakovic et al. 2025a). In this case, stress relaxation induced by the remodeling of adhesion contacts is the main cause of energy dissipation (Pajic-Lijakovic and Milivojevic 2025). Viscoelasticity of epithelial multicellular systems caused by collective cell migration for $\:{n}_{e}\le\:{n}_{conf}$ satisfies the following conditions:

- Cell stress can relax under the condition of constant strain (Khalilgharibi et al. 2019). Stress relaxation is exponential (Marmottant et al. 2009) indicating linear viscoelastic solid behaviour.
- The ability of strain to relax under the condition of constant (or zero) stress pointed to a particular linear constitutive model, i.e., the Zener model for viscoelastic solids (Pajic-Lijakovic et al. 2024).

The anisotropic Zener model is developed by incorporating the concept of additive isotropic and supplementary anisotropic contributions to stress, modifying the model introduced by Pajic-Lijakovic et al. (2024) for the isotropic scenario. The model is presented as a function of the degree of anisotropy as:

3 $$\:{\stackrel{\sim}{\boldsymbol{\sigma\:}}}_{i}+{\tau\:}_{R}{\dot{\stackrel{\sim}{\boldsymbol{\sigma\:}}}}_{i}={{\stackrel{\sim}{\boldsymbol{\sigma\:}}}^{0}}_{i}\left({\stackrel{\sim}{\boldsymbol{\epsilon\:}}}_{\boldsymbol{i}},{\dot{\stackrel{\sim}{\boldsymbol{\epsilon\:}}}}_{\boldsymbol{i}}\right)+\alpha\:\varDelta\:{\stackrel{\sim}{\boldsymbol{\sigma\:}}}_{i}\left({\stackrel{\sim}{\boldsymbol{\epsilon\:}}}_{\boldsymbol{i}},{\dot{\stackrel{\sim}{\boldsymbol{\epsilon\:}}}}_{\boldsymbol{i}}\right)$$

where $\:i\equiv\:S,N$, $\:S$ is the shear component $\:{\sigma\:}_{xy}$ and $\:N$ are the normal components $\:{\sigma\:}_{xx}$ and $\:{\sigma\:}_{yy}$, $\:{\stackrel{\sim}{\boldsymbol{\sigma\:}}}_{i}$ is the cell stress (shear or normal), $\:\dot{\stackrel{\sim}{\boldsymbol{\sigma\:}}}$ is the rate of stress change equal to $\:{\dot{\stackrel{\sim}{\boldsymbol{\sigma\:}}}}_{i}=\frac{d}{dt}{\stackrel{\sim}{\boldsymbol{\sigma\:}}}_{i}$, $\:{{\stackrel{\sim}{\boldsymbol{\sigma\:}}}^{0}}_{i}$ is the isotropic stress equal to $\:{{\stackrel{\sim}{\boldsymbol{\sigma\:}}}^{0}}_{i}\left({\stackrel{\sim}{\boldsymbol{\epsilon\:}}}_{\boldsymbol{i}},{\dot{\stackrel{\sim}{\boldsymbol{\epsilon\:}}}}_{\boldsymbol{i}}\right)={E}_{\boldsymbol{i}}{\stackrel{\sim}{\boldsymbol{\epsilon\:}}}_{\boldsymbol{i}}+{\eta\:}_{\boldsymbol{i}}{\dot{\stackrel{\sim}{\boldsymbol{\epsilon\:}}}}_{\boldsymbol{i}}$, $\:{E}_{\boldsymbol{i}}\equiv\:{E}_{\boldsymbol{s}}$ is shear modulus, $\:{E}_{\boldsymbol{i}}\equiv\:{E}_{\boldsymbol{N}}$ is the Young’s modulus, $\:{\stackrel{\sim}{\boldsymbol{\epsilon\:}}}_{\boldsymbol{i}}$ is the strain such that the shear strain is equal to $\:{\stackrel{\sim}{\boldsymbol{\epsilon\:}}}_{\boldsymbol{s}}=\frac{1}{2}\left(\overrightarrow{\nabla\:}\overrightarrow{\boldsymbol{u}}+{\overrightarrow{\nabla\:}\overrightarrow{\boldsymbol{u}}}^{\boldsymbol{T}}\right)$ and the normal strain is $\:{\stackrel{\sim}{\boldsymbol{\epsilon\:}}}_{\boldsymbol{N}}=\overrightarrow{(\nabla\:}\cdot\:\overrightarrow{\boldsymbol{u}})\stackrel{\sim}{\boldsymbol{I}}$, $\:\overrightarrow{\boldsymbol{u}}$ is the cell local displacement field, $\:\stackrel{\sim}{\boldsymbol{I}}$ is the unit tensor, $\:{\eta\:}_{\boldsymbol{i}}\equiv\:{\eta\:}_{\boldsymbol{s}}$ is the shear viscosity, and $\:{\eta\:}_{\boldsymbol{i}}\equiv\:{\eta\:}_{\boldsymbol{N}}$ is the bulk viscosity, $\:{\tau\:}_{R}$ is the stress relaxation time, and $\:\varDelta\:{\stackrel{\sim}{\boldsymbol{\sigma\:}}}_{i}$ is the anisotropic contribution to stress equal to $\:\varDelta\:{\stackrel{\sim}{\boldsymbol{\sigma\:}}}_{i}\left({\stackrel{\sim}{\boldsymbol{\epsilon\:}}}_{\boldsymbol{i}},{\dot{\stackrel{\sim}{\boldsymbol{\epsilon\:}}}}_{\boldsymbol{i}}\right)={\stackrel{\sim}{\boldsymbol{\sigma\:}}}_{i}-\frac{1}{d}tr\left({\stackrel{\sim}{\boldsymbol{\sigma\:}}}_{i}\right)\stackrel{\sim}{\boldsymbol{I}}$, and $\:\alpha\:$ is the degree of anisotropy, which will be discussed in next section. In accordance with the fact that cells are most sensitive to shear stress, as discussed above, we will present Eq. 3 for the shear stress component, while noting that similar equations can be formulated for all stress components. Consequently, the shear stress as a function of all strain components and the degree of anisotropy in the form of the Zener model can be expressed as:

4 $$\begin{aligned}\:{\sigma\:}_{xy}\left(x,y,t,\tau\:\right)+{\tau\:}_{R}{\dot{\sigma\:}}_{xy}&={E}_{s}{\epsilon\:}_{xy}+{\eta\:}_{s}{\dot{\epsilon\:}}_{xy}\\&\quad+\alpha\:\left[{A}_{1}{\epsilon\:}_{xx}+{A}_{2}{\epsilon\:}_{yy}+{B}_{1}{\dot{\epsilon\:}}_{xx}+{B}_{2}{\dot{\epsilon\:}}_{yy}\right]\end{aligned}$$

where $\:{\sigma\:}_{xy}\left(x,y,t,\tau\:\right)$ is the shear stress, $\:t$ is a short timescale of minutes, $\:\tau\:$ is a long timescale of hours, $\:{\dot{\sigma\:}}_{xy}$ is the rate of shear stress change equal to $\:{\dot{\sigma\:}}_{xy}=\frac{d{\sigma\:}_{xy}}{dt}$, $\:{\epsilon\:}_{xy}$, $\:{\epsilon\:}_{xx}$, and $\:{\epsilon\:}_{yy}$ are shear and normal strain components in *x*- and *y*-directions, respectively, $\:{\dot{\epsilon\:}}_{xy}$ is the rate of shear strain change equal to $\:{\dot{\epsilon\:}}_{xy}=\frac{d{\epsilon\:}_{xy}}{d\tau\:}$, $\:{\dot{\epsilon\:}}_{xx}$ is the rate of change the normal strain in *x*-direction equal to $\:{\dot{\epsilon\:}}_{xx}=\frac{d{\epsilon\:}_{xx}}{d\tau\:}$, $\:{\dot{\epsilon\:}}_{yy}$ is the rate of change of the normal strain in the *y*-direction, $\:{E}_{s}$ is the shear modulus of elasticity, $\:{\eta\:}_{s}$ is the shear viscosity, and the parameters $\:{A}_{1}$ and $\:{A}_{2}$ represent the additional elastic moduli and quantify the elastic contribution to the shear stress caused by change in the normal strain components, while the parameters $\:{B}_{1}$ and $\:{B}_{2}$ represents the additional viscosity terms and quantify the viscous contribution to the shear stress caused by change in the rate of normal strain components. Similar expressions apply to the normal stress components as well.

**Fig. 4:**
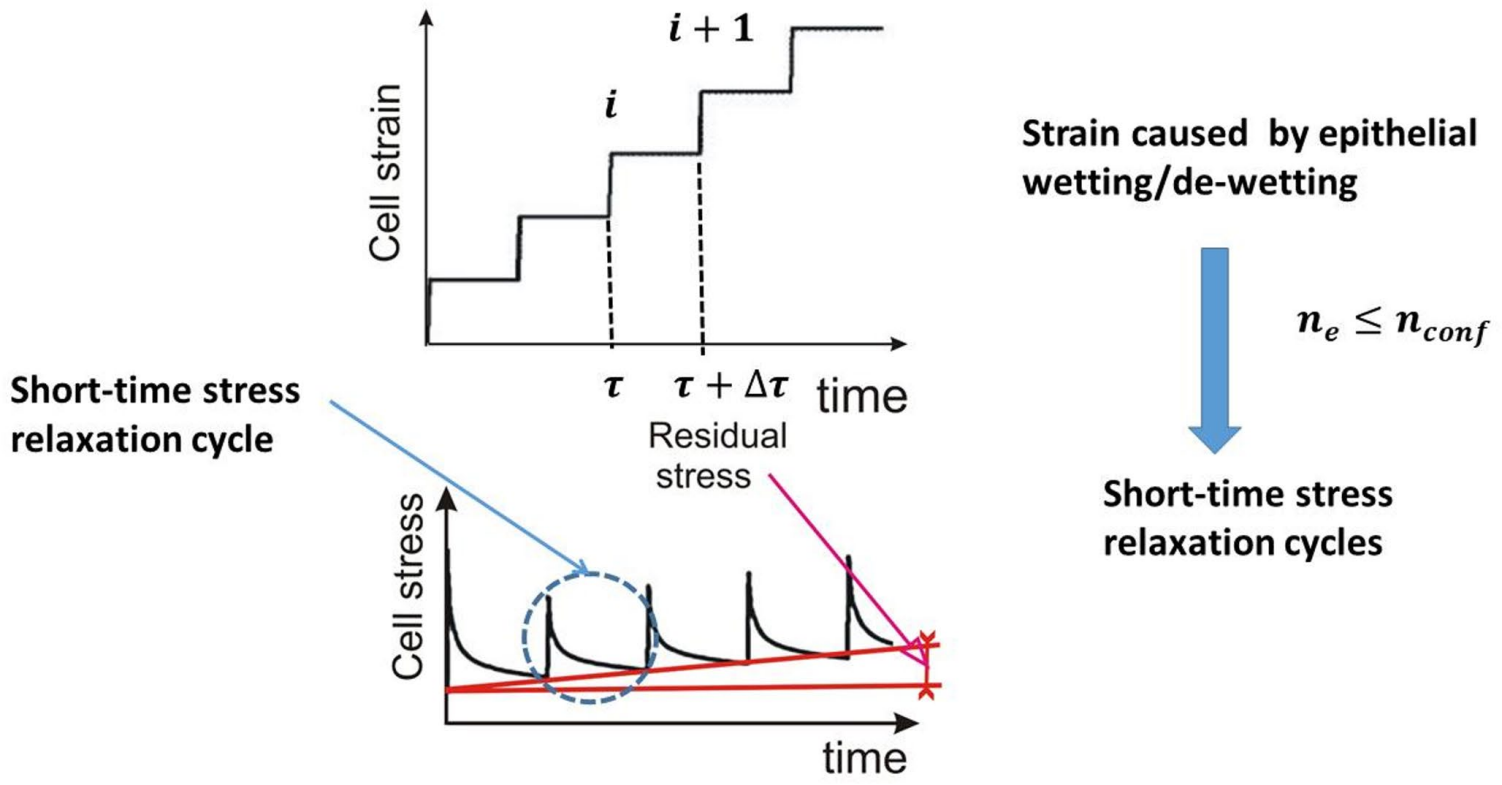
The generation of mechanical stress takes place during successive short-time stress relaxation cycles that occur during collective cell migration, specifically when the cell packing density$\:\:{n}_{e}$ is less than or equal to $\:{n}_{conf}$, resulting in a progressive rise in the residual stress

The stress relaxes over minutes through successive short-time relaxation cycles under constant strain per cycle, while strain and residual stress change over hours as was shown in Fig. 4:

Accordingly, the parameters $\:{A}_{1}$, $\:{A}_{2}$, $\:{B}_{1}$, and $\:{B}_{2}$ from Eq. 4 can be estimated experimentally within migrating epithelial monolayers by following a systematic procedure:

(i). Measurement of the long-term change of shear stress $\:{{\sigma\:}_{xy}}^{r}$ (i.e., the residual shear stress) and the degree of anisotropy $\:\alpha\:$;
(ii). Elastic modulus $\:{E}_{s}$ should be measured;
(iii). At $\:\tau\:={\tau\:}_{0}$, after the first short-time relaxation cycle under constant strain components per cycle $\:{\epsilon\:}_{xy0}$, $\:{\epsilon\:}_{xx0}$, and $\:{\epsilon\:}_{yy0}$, the residual shear stress is equal to:

$$\:{{\sigma\:}_{xy0}}^{r}={E}_{s}{\epsilon\:}_{xy0}+{\alpha\:}_{0}\left[{A}_{1}{\epsilon\:}_{xx0}+{A}_{2}{\epsilon\:}_{yy0}\right]$$

(iv). At $\:{\tau\:}_{1}={\tau\:}_{0}+\varDelta\:\tau\:$, the second short-time stress relaxation cycle is finished, while the new value of the shear residual stress is equal to:

$$\:{{\sigma\:}_{xy1}}^{r}={E}_{s}{\epsilon\:}_{xy1}+{\alpha\:}_{1}\left[{A}_{1}{\epsilon\:}_{xx1}+{A}_{2}{\epsilon\:}_{yy1}\right]$$

(v). Parameters $\:{A}_{1}$ and $\:{A}_{2}$ can be estimated from the values of the shear stress $\:{{\sigma\:}_{xy0}}^{r}$ and $\:{{\sigma\:}_{xy1}}^{r}$ and compared with values calculated from the next set of values of the shear residual stress component.
(vi). Parameters $$\:{B}_{1}$$ and $$\:{B}_{2}$$ can be estimated by correlating the values of the residual stress after two successive stress-relaxation cycles, i.e., $$\:{{\sigma\:}_{xy0}}^{r}$$ and $$\:{{\sigma\:}_{xy1}}^{r}$$. This interrelationship can be formulated as: $$\:{{\sigma\:}_{xy1}}^{r}={{\sigma\:}_{xy0}}^{r}+\frac{{\alpha\:}_{0}+{\alpha\:}_{1}}{2}$$$$\left[{B}_{1}\frac{{\epsilon\:}_{xx1}-{\epsilon\:}_{xx0}}{\varDelta\:\tau\:}+{B}_{1}\frac{{\epsilon\:}_{yy1}-{\epsilon\:}_{yy0}}{\varDelta\:\tau\:}\right]$$.

This procedure of calculation should be repeated for the measured values of shear residual stress vs. time. Pairs of successive stress relaxation cycles enable calculation of parameters $\:{A}_{1}$, $\:{A}_{2}$, $\:{B}_{1}$, and $\:{B}_{2}$. The distribution of parameters can be obtained as the result of this calculation accompanied by their average values.

An increase in compressive stress, accompanied by an increase in cell packing density within migrating epithelial collectives: (i) suppresses the relaxation of stress and (ii) induces a decrease in the degree of anisotropy. For the cell packing density $\:{n}_{e}>{n}_{conf}$ the mechanism of cell migration changes from convective to diffusive (Pajic-Lijakovic and Milivojevic, 2021b). When cell packing density become high enough, altered cell-cell interactions caused by contact inhibition of locomotion, lead to a pronounced increase in the stress relaxation time such that $\:{\tau\:}_{R}\to\:\infty\:$ and the degree of anisotropy $\:\alpha\:\to\:0$. In this case, the constitutive model described by Eqs. 3–4, should be transformed to the Kelvin-Voigt model suitable for describing isotropic wetting/de-wetting of multicellular domains. It is expressed as (Pajic-Lijakovic and Milivojevic, 2021b):

5 $$\:{\stackrel{\sim}{\boldsymbol{\sigma\:}}}_{i}={E}_{\boldsymbol{i}}{\stackrel{\sim}{\boldsymbol{\epsilon\:}}}_{\boldsymbol{i}}+{\eta\:}_{\boldsymbol{i}}{\dot{\stackrel{\sim}{\boldsymbol{\epsilon\:}}}}_{\boldsymbol{i}}$$

In this case, the mechanism of energy dissipation is caused by the perturbation of cell alignment, which occurs over hours. Given that anisotropic cell migration leads to increased mechanical stress, it is necessary to explore potential cellular strategies for safeguarding against stress, and particularly shear stress. 

## The distribution of the degree of anisotropy

The main characteristics of migrating epithelial collectives lie in the inhomogeneous distributions of relevant physical parameters such as: the degree of anisotropy, cell packing density, velocity, corresponding strain, cell mechanical stress, and spreading factor (Serra-Picamal et al. 2012; Nnetu et al. 2013; Notbohm et al. 2016; Tlili et al. 2018; Pajic-Lijakovic et al. 2024). Consequently, a migrating epithelial collective can be treated as an ensemble of multicellular, mesoscopic domains characterized by homogeneous distributions of these physical parameters within each domain (Pajic-Lijakovic and Milivojevic, 2021b). Domains are unstable. They exist for some period of time and then lose their identity through interactions among the domains. These interactions lead to an increase in mechanical stress, which has a feedback impact on the state of cell-cell and cell-matrix adhesion contacts, and of cell contractility. Such interactions may give rise to phenomena such as: cell jamming, live cell extrusion, or the epithelial-to-mesenchymal transition. The lifetime of a domain is typically on a timescale of hours, which corresponds to collective cell migration and cell repolarisation caused by head-on interactions (Notbohm et al. 2016). The degree of anisotropy within a domain can be related to the relative fractions of aligned cells in the direction of migration and in the perpendicular direction:

6 $$\:\alpha\:\left(\tau\:\right)=\frac{{y}_{0}\left(r,\tau\:\right)-{y}_{\pi\:/2}\left(r,\tau\:\right)}{{y}_{0}\left(r,\tau\:\right)}$$

where $\:r\left(x,y\right)$ is the local coordinate, $\:\tau\:$ is a timescale of hours, which corresponds to that of collective cell migration, $\:{y}_{0}$ is the fraction of cells oriented in the direction of migration for $\:\theta\:=0$ and is equal to $\:{y}_{0}\left(r,\tau\:\right)={\int\:}_{0}^{\varDelta\:\theta\:}\rho\:\left(r,\theta\:,\tau\:\right)d\theta\:$, $\:\rho\:\left(r,\theta\:,\tau\:\right)$ is the probability density function for finding cell at a position $\:r$ and polarity $\:\theta\:$ at time $\:\tau\:$.

of cell orientation, and $\:{y}_{\pi\:/2}$ is the fraction of cells oriented in the perpendicular direction for $\:\theta\:=\frac{\pi\:}{2}$, equal to $\:{y}_{\pi\:/2}\left(r,\tau\:\right)={\int\:}_{\pi\:/2}^{\varDelta\:\theta\:}\rho\:\left(r,\theta\:,\tau\:\right)d\theta\:$, while $\:\varDelta\:\theta\:\ll\:\frac{\pi\:}{2}$. The degree of anisotropy, $\:\alpha\:$ is in the range $\:\alpha\:\in\:\left[\mathrm{0,1}\right]$. Isotropic cell migration is quantified by the degree of anisotropy, ranging from zero, i.e., $\:\alpha\:=0$, to a maximum value of $\:\alpha\:=1$.

The time-evolution of the probability density function $\:\rho\:\left(\theta\:,\tau\:\right)$ can be expressed in the form of a Fokker-Planck equation as (Patel et al. 2025):

7 $$\:\frac{\partial\:\rho\:\left(r,\theta\:,\tau\:\right)}{\partial\:\tau\:}+\overrightarrow{\nabla\:}\left({v}_{o}\overrightarrow{\boldsymbol{Q}}\rho\:\right)=\frac{1}{{\tau\:}_{a}}\frac{\partial\:}{\partial\:\theta\:}\left(\rho\:\frac{\partial\:}{\partial\:\theta\:}{U}_{\theta\:}\right)+{D}_{R}\frac{{\partial\:}^{2}}{\partial\:{\theta\:}^{2}}\rho\:$$

where $$\:{D}_{R}$$ is the rotation diffusion coefficient, which depends on the cell shear stress component, i.e., $$\:{D}_{R}={D}_{R}\left({\sigma\:}_{xy}\right)$$, $$\:\overrightarrow{\boldsymbol{Q}}$$ is the orientational vector equal to $$\:\overrightarrow{\boldsymbol{Q}}=cos\theta\:{\overrightarrow{\:\boldsymbol{e}}}_{\boldsymbol{x}}+\:sin\theta\:{\overrightarrow{\:\boldsymbol{e}}}_{\boldsymbol{y}}$$ (where $$\:{\overrightarrow{\:\boldsymbol{e}}}_{\boldsymbol{x}}$$ and $$\:{\overrightarrow{\:\boldsymbol{e}}}_{\boldsymbol{y}}$$ are unit vectors in *x*- and *y*-directions) (Smeets et al. 2016; Lin et al. 2018), $$\:{v}_{o}$$ is the self-propulsion speed, and $$\:{\tau\:}_{a}$$ is the relaxation time of cell alignment in the direction of migration, which depends on the cell packing density $$\:{n}_{e}$$ and effective temperature $$\:{T}_{eff}$$ i.e., $$\:{\tau\:}_{a}\sim({n}_{e},{T}_{eff}^{-1})$$, and $$\:{U}_{\theta\:}\left(r,\theta\:,\tau\:\right)$$ is the dimensionless alignment potential. While (Smeets et al. 2016) considered the contribution to the alignment potential caused by contact inhibition of locomotion, (Lin et al. 2018) considered two contributions to the potential $$\:{U}_{\theta\:}\left(r,\theta\:,\tau\:\right)$$: (i) local alignment to velocity direction $$\:{U}_{LA}=-J{\int\:}_{neigh}^{}d{r}^{{\prime\:}}{\int\:}_{0}^{2\pi\:}cos\left({\theta\:}^{v}-{\theta\:}^{{\prime\:}}\right)\rho\:\left({r}^{{\prime\:}},{\theta\:}^{{\prime\:}},\tau\:\right)d{\theta\:}^{{\prime\:}}$$ and (ii) alignment caused by contact inhibition of locomotion, which depends on the distance between cells $$\:{U}_{CIL}=-K{\int\:}_{neigh}^{}d{r}^{{\prime\:}}{\int\:}_{0}^{2\pi\:}cos\left({\theta\:}^{r}-{\theta\:}^{{\prime\:}}\right)\rho\:\left({r}^{{\prime\:}},{\theta\:}^{{\prime\:}},\tau\:\right)d{\theta\:}^{{\prime\:}}$$, i.e., $$\:{U}_{\theta\:}={U}_{LA}+{U}_{CIL}$$ (Lin et al. 2018) (where $$\:{\theta\:}^{v}$$ is the velocity direction $$\:{\theta\:}^{v}=\mathrm{a}\mathrm{r}\mathrm{g}\left({\overrightarrow{\boldsymbol{v}}}_{\boldsymbol{r}}\right)$$, $$\:{\overrightarrow{\boldsymbol{v}}}_{\boldsymbol{r}}$$ is the average velocity of the group of cells located at r, and $$\:{\theta\:}^{r}$$ is the argument of the direction between cells $$\:i$$ and $$\:j$$$$\:{\theta\:}^{r}=\mathrm{a}\mathrm{r}\mathrm{g}\left({\overrightarrow{\boldsymbol{r}}}_{\boldsymbol{i}\boldsymbol{j}}\right)$$, while $$\:J$$ and $$\:K$$ are dimensionless parameters that quantify the strengths of these two types of interaction). While local alignment to the velocity direction dominantly influences cell rearrangement under lower cell packing density for $$\:{n}_{e}<{n}_{conf}$$, the alignment caused by contact inhibition of locomotion significantly influences cell rearrangement under higher cell packing density for $$\:{n}_{e}>{n}_{conf}$$ (where $$\:{n}_{conf}$$ is the cell packing density in the confluent state). (Notbohm et al. 2016) considered the migration of confluent Madin-Darbvy canine kidney type II cell monolayers and pointed out that cell alignment to velocity direction was not observed. An increase in the relaxation time of cell alignment, caused by an increase in cell packing density results in a decrease in the degree of anisotropy. However, an increase in cell mobility quantified by effective temperature decreases the relaxation time. The concept of effective temperature has been utilized to examine the rearrangement of diverse thermodynamic systems, ranging from those close to equilibrium to those far from it, including glasses, sheared fluids, and granular systems (Casas-Vazquez and Jou, 2003). Pajic-Lijakovic and Milivojevic (2021a) applied this concept to the long-time cell rearrangement of dense cellular systems. In this case, the effective temperature results from cell migration and is expressed as: $$\:{\left({k}_{B}{T}_{eff}\right)}^{1/2}\sim\langle\parallel{\overrightarrow{\boldsymbol{v}}}_{\boldsymbol{c}}\parallel\rangle$$ (where $$\:\parallel\langle{\overrightarrow{\boldsymbol{v}}}_{\boldsymbol{c}}\parallel\rangle$$ is the average cell speed) (Casas-Vazquez and Jou 2003; Pajic-Lijakovic and Milivojevic 2021a). The more-mobile cells easily re-establish the ordered trend of cell migration. The orientation vector $$\:\overrightarrow{\boldsymbol{Q}}$$ tends to align with strain. The degree of anisotropy influences cell spreading and the generation of mechanical stress.

The main factor contributing to a reduction in the level of anisotropy is the interactions between cells. Cell-cell interactions are intensified by an increase in cell packing density under constant cell speed. Otherwise, an increase in cell mobility under constant cell packing density reduces cell-cell interactions. Consequently, the degree of anisotropy: (i) increases with the gradient of some external field such as concentration of nutrients, the electrostatic field, matrix stiffness, the epithelial spreading factor and others, (ii) decreases with an increase in cell packing density and (iii) increases with effective temperature. It can be expressed as:

8 $$\:\frac{d\alpha\:}{d\tau\:}={\left(\frac{\partial\:\alpha\:}{\partial\:\overrightarrow{\boldsymbol{\phi\:}}}\right)}_{{n}_{e},{T}_{eff}}\frac{d\overrightarrow{\boldsymbol{\phi\:}}}{d\tau\:}-{\left(\frac{\partial\:\alpha\:}{\partial\:{n}_{e}}\right)}_{\overrightarrow{\boldsymbol{\phi\:},}{T}_{eff}}\frac{d{n}_{e}}{d\tau\:}+{\left(\frac{\partial\:\alpha\:}{\partial\:{T}_{eff}}\right)}_{\overrightarrow{\boldsymbol{\phi\:},}{n}_{e}}\frac{d{T}_{eff}}{d\tau\:}$$

where $\:\overrightarrow{\phi\:}=\overrightarrow{\nabla\:}\chi\:$, and $\:\chi\:$ is the external scalar field. An increase in cell mobility perturbs the cell alignment resulting in a decrease in the degree of anisotropy.

Multicellular domains near the edge of the monolayer are more active and migrate more efficiently than domains within the central region (Serra-Picamal et al. 2012). The degree of anisotropy in these domains near the monolayer edge is higher and decreases towards that of the domains in the central region. Cells tend to migrate from regions of lower anisotropy (more isotropic) to regions of high anisotropy (more aligned) by following the gradient of cell packing density (Lin et al. 2025). Intensive cell-cell interactions in an overcrowded environment can perturb cell alignment, resulting in more isotropic cell migration (Pajic-Lijakovic et al. 2024). These interactions are related to the inhomogeneous wetting/de-wetting of epithelial monolayers on substrate matrices.

## The impact of the degree of anisotropy on the generation of strain during epithelial wetting/de-wetting

Wetting/de-wetting of the domains causes the generation of uni-axial/bi-axial extensional/compressional strain depending on the degree of anisotropy (Pajic-Lijakovic et al. 2024). Boundary conditions can be established for: (i) the isotropic case $\:\alpha\:=0$ and (ii) the anisotropic case $\:\alpha\:=1$. Extension/compression of isotropic domains is equal in all directions and can be treated as biaxial. However, extension/compression of anisotropic domains with the degree of anisotropy $\:\alpha\:=1$ is rather uniaxial. Consequently, two scenarios can be considered:

- In isotropic domains where $\:\alpha\:=0$, the normal strain component in the *x*-direction, denoted as $\:{\epsilon\:}_{xx}$, is equivalent to the normal strain component in the *y*-direction, represented as $\:{\epsilon\:}_{yy}$, such that $\:{\epsilon\:}_{yy}={\epsilon\:}_{xx}$. These strain components can be categorized as: (i) extensional, where $\:{\epsilon\:}_{yy}>0$ and $\:{\epsilon\:}_{xx}>0$, indicating an active wetting condition, or (ii) compressive, where $\:{\epsilon\:}_{yy}<0$ and $\:{\epsilon\:}_{xx}<0$, indicating a de-wetting condition.
- In the context of anisotropic domains ($\:\alpha\:=1$), it is observed that when cells migrate along the *x*-direction, the relationship $\:{\epsilon\:}_{yy}=-\nu\:{\epsilon\:}_{xx}$ holds, where *ν* represents the viscoelastic, space-time-dependent Poisson’s ratio (Pajic-Lijakovic et al. 2025b). The average Piosson’s ratio of Madin-Darby Canine Kidney-MDCK and HeLa epithelial monolayers is $\:\nu\:\sim0.77$ (Moisdon et al. 2022). When the Poisson’s ratio $\:\nu\:>0.5$, extension in the *x*-direction induces compression in *y*-direction and vice versa. It means that: (i) wetting can be characterised by $\:{\epsilon\:}_{xx}>0$ and $\:{\epsilon\:}_{yy}<0$ and (ii) de-wetting can be characterised by $\:{\epsilon\:}_{xx}<0$ and $\:{\epsilon\:}_{yy}>0$. While cells perform active wetting/de-wetting in the direction of cell migration, passive wetting/de-wetting occurs in the perpendicular direction.

Both scenarios can be incorporated in terms of the following relationship between two normal strain components, expressed as:

9 $$\:{\epsilon\:}_{yy}\left(\alpha\:,\nu\:\right)=\left(1-\alpha\:-\alpha\:\nu\:\right){\epsilon\:}_{xx}$$

Besides normal strain components, a shear strain component $\:{\epsilon\:}_{xy}$ is generated within the domains and along the border between domains. All strain components contribute to the generation of every component of mechanical stress depending on the degree of anisotropy.

## Cell strategies to decrease undesirable shear stress

**Fig. 5:**
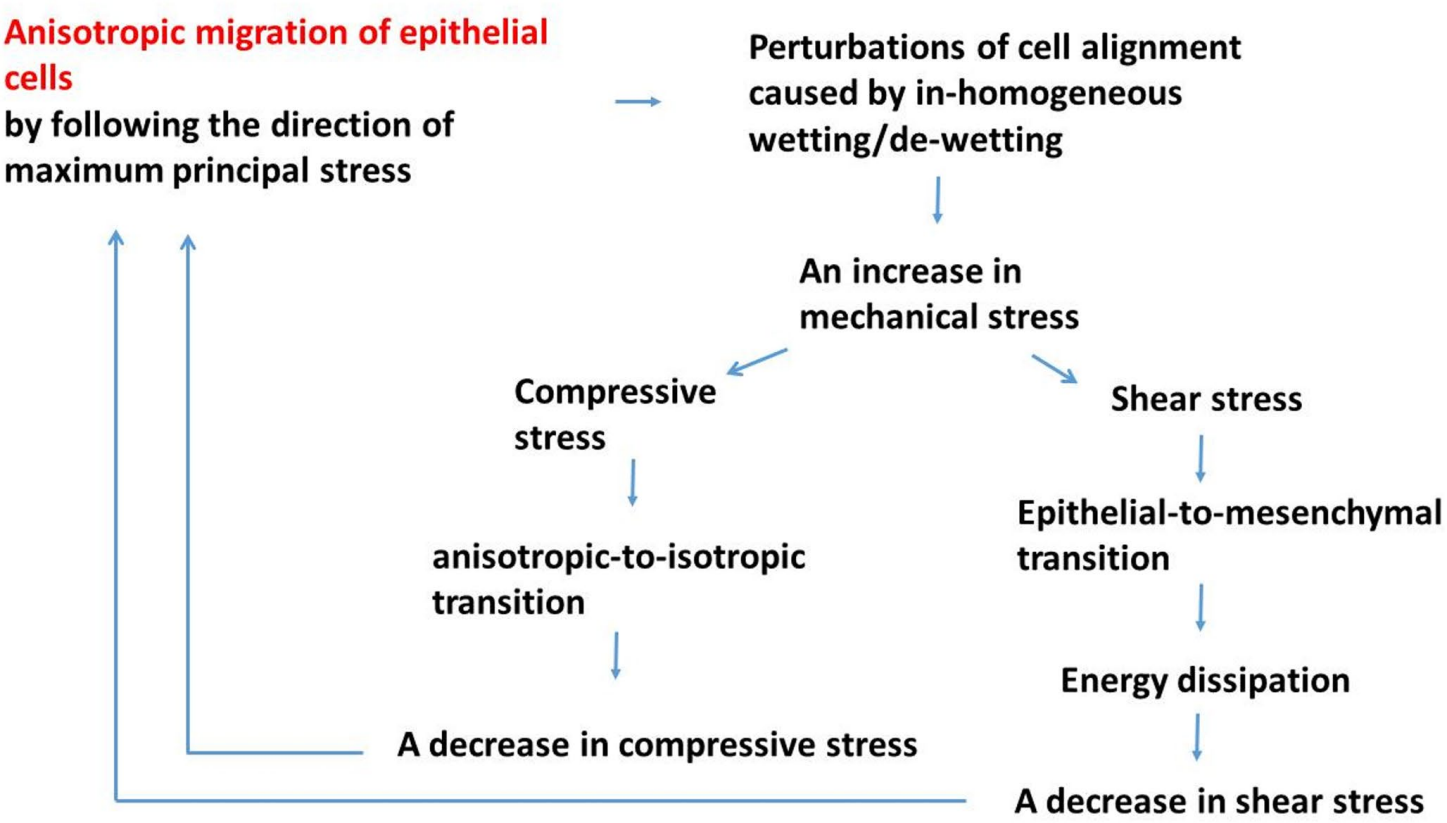
Schematic presentations of cell strategies on decreasing shear stress

Collective cell migration generates mechanical stress. Cells develop a mechanism to control its magnitude, to some extent, by remodelling their cell cell-adhesion contacts (Iyer et al. 2019; Barriga and Mayor 2019) and by changing the degree of anisotropy caused by cell-cell interactions (Roycraft and Mayoer 2016; Pajic-Lijakovic et al. 2024). Particular emphasis is placed on the reduction of shear stress, aligning with the understanding that cells exhibit heightened sensitivity to shear stress (Espina et al. 2023). Several cell strategies to avoid and minimize undesirable shear stress have been discussed: (i) cell migration along the maximum principal stress by retaining the degree of anisotropy (Trepat and Fredberg 2011), (ii) the anisotropic-to-isotropic transition caused by an increase in compressive stress and cell packing density (Pajic-Lijakovic and Milivojevic 2021b), and (iii) energy dissipation caused by the weakening of cell-cell adhesion contacts induced by the epithelial-to-mesenchymal transition (Barriga and Mayor, 2019; Pajic-Lijakovic and Milivojevic 2025). Cell strategies on decreasing shear stress are presented schematically in Fig. 5:

- If cells retain directional migration by keeping a high degree of anisotropy, they can minimize shear stress and mechanical resistance by migrating along the direction of maximum principal stress. The latter corresponds to the direction of greatest tensile (or least-compressive) stress and can be expressed as:

10 $$\:\stackrel{\sim}{\boldsymbol{\sigma\:}}\cdot\:\overrightarrow{\boldsymbol{Q}}={\sigma\:}_{1}\cdot\:\overrightarrow{\boldsymbol{Q}}$$

where $\:{\sigma\:}_{1}$ represents the one of the eigenvalues of the stress tensor, i.e. the maximum principal stress. By following the direction of maximum principal stress, cells can minimise the generation of shear stress. This is a central idea in the concept known as plithotaxis (Trepat and Fredberg 2011). However, in confined environments (e.g., narrow channels or crowded tissues), cells are forced to deviate from stress-aligned migration. In dynamic tissues, principal stress directions may be spatially varying or rapidly changing, making it impractical for a cell to continuously reorient (Saw et al. 2017). This variation can cause an increase in the shear stress. What could be the cell response to this increase?

- Cells can undergo the epithelial-to-mesenchymal transition (EMT) as a mechanism to mitigate the accumulation of shear stress by maintaining some degree of anisotropy. Research indicates that a shear stress of just 0.14 Pa can trigger EMT in Hep-2 cells (Liu et al. 2016). Furthermore, a shear stress of 0.3 Pa is sufficient to induce EMT in epithelial ovarian cancer (Rizvi et al. 2013). In contrast, very much greater compressive stress is required to initiate EMT, with a partial EMT being induced by a compressive stress of approximately 600 Pa (Tse et al. 2012). While epithelial cells establish strong E-cadherin-mediated cell-cell adhesion contacts leading to the accumulation of mechanical stress, mesenchymal cells establish weak N-cadherin-mediated cell-cell adhesion contacts resulting in the dissipation of cell residual stress (Barriga and Mayore, 2019). Migration of mesenchymal cells induces intensive energy dissipation leading to a decrease of previously accumulated shear stress. In contrast to epithelial collectives, which behave as viscoelastic solids, migrating mesenchymal collectives has been treated as viscoelastic liquids (Pajic-Lijakovic et al. 2025a). A suitable constitutive model for anisotropic movement of mesenchymal cells could be expressed from Eq. 6 by supposing that the elastic terms are much smaller than the dissipative, viscous term and can be neglected. The form of the model proposed by Pajic-Lijakovic et al. (2025a) in this case corresponds to the modified Maxwell model for the anisotropic migration of mesenchymal cells expressed as:

11 $$\:{\stackrel{\sim}{\boldsymbol{\sigma\:}}}_{i}+{\tau\:}_{R}{\dot{\stackrel{\sim}{\boldsymbol{\sigma\:}}}}_{i}={{\stackrel{\sim}{\boldsymbol{\sigma\:}}}^{0}}_{i}\left({\dot{\stackrel{\sim}{\boldsymbol{\epsilon\:}}}}_{\boldsymbol{i}}\right)+\alpha\:\varDelta\:{\stackrel{\sim}{\boldsymbol{\sigma\:}}}_{i}\left({\dot{\stackrel{\sim}{\boldsymbol{\epsilon\:}}}}_{\boldsymbol{i}}\right)$$

where the isotropic contribution to stress is expressed as $\:{{\stackrel{\sim}{\boldsymbol{\sigma\:}}}^{0}}_{i}\left({\dot{\stackrel{\sim}{\boldsymbol{\epsilon\:}}}}_{\boldsymbol{i}}\right)={\eta\:}_{\boldsymbol{i}}{\dot{\stackrel{\sim}{\boldsymbol{\epsilon\:}}}}_{\boldsymbol{i}}$. The shear stress component is influenced by the rate at which all strain components vary, as expressed by the following equation:

12 $$\:{\sigma\:}_{xy}\left(x,y,t,\tau\:\right)+{\tau\:}_{R}{\dot{\sigma\:}}_{xy}={\eta\:}_{s}{\dot{\epsilon\:}}_{xy}+\alpha\:\left[{B}_{1}{\dot{\epsilon\:}}_{xx}+{B}_{2}{\dot{\epsilon\:}}_{yy}\right]$$

The pronounced mobility of mesenchymal cells in comparison to epithelial cells influences physical parameters such as the stress relaxation time and the degree of anisotropy. The relaxation time of mesenchymal cells represented as $\:{\tau\:}_{R}^{m}$ (from Eq. 11) is lower than the relaxation time of epithelial cells denoted as $\:{\tau\:}_{R}^{e}$ (from Eq. 4), i.e., $\:{\tau\:}_{R}^{e}>{\tau\:}_{R}^{m}$. The inertness of epithelial cells, quantified by their longer relaxation time, arises from the fact that these cells establish strong cell-cell adhesion contacts. The degree of anisotropy of mesenchymal cells $\:{\alpha\:}^{m}$ is lower than the degree of anisotropy of epithelial cells $\:{\alpha\:}^{e}$, i.e., $\:{\alpha\:}^{m}<{\alpha\:}^{e}$ for the same cell packing density, which is lower than or equal to the cell packing density under the confluent state. The strain rate of mesenchymal cells $\:{\dot{\stackrel{\sim}{\boldsymbol{\epsilon\:}}}}^{\boldsymbol{m}}$ caused by collective cell migration is higher than the strain rate of epithelial cells $\:{\dot{\stackrel{\sim}{\boldsymbol{\epsilon\:}}}}^{\boldsymbol{e}}$, i.e., $\:{\dot{\stackrel{\sim}{\boldsymbol{\epsilon\:}}}}^{\boldsymbol{m}}>{\dot{\stackrel{\sim}{\boldsymbol{\epsilon\:}}}}^{\boldsymbol{e}}$.

- When cells retain the epithelial phenotype, an interplay between shear and compressive stress is needed to perturb cell alignment and induce the anisotropic-to-isotropic cell state transition for a cell packing density higher than that of the confluent state (Saw et al. 2017). An increase in compressive stress induces an increase in cell packing density, leading to intensive cell head-on interactions, which results in contact inhibition of locomotion (CIL) (Pajic-Lijakovic et al. 2024) through a weakening of cell-cell and cell-matrix adhesion contacts (Roycraft and Mayor 2016). When the time between two collisions $\:{t}_{c}$ is longer than the time required for cell re-polarisation $\:{t}_{rp}$, cells have enough time to re-establish strong cell-cell adhesion contacts and migrate in the opposite direction by perturbing cell alignment (Pajic-Lijakovic et al. 2025). (Notbohm et al. 2016) indicated that the mean repolarization duration during the reorganization of confluent MDCK cell monolayers is $\:1.28\:h$. Shear stress induces single-cell rotation by additionally perturbing the direction of migration of re-polarised cells (Saw et al. 2017).

These scenarios suggested that priorities of migrating epithelial collectives are to retain a higher degree of anisotropy and to follow the maximum principal stress direction. However, inhomogeneous wetting/de-wetting can perturb the direction of cell migration by increasing a cell’s exposure to shear stress. In this case, two scenarios are possible depending primarily on the magnitude of the cell compressive stress (Saw et al. 2017; Pajic-Lijakovic et al. 2024). Increases in compressive stress and cell packing density induce the transition from anisotropic-to-isotropic cell migration. However, an increase in shear stress under the same cell packing density leads to the epithelial-to-mesenchymal cell state transitions. Both outcomes result in a decrease in cell shear stress.

## Conclusion

Anisotropy is the one of the main physical aspects of efficient cell migration. At the same time, anisotropy is the one of the main causes of intensive accumulation of mechanical stress within migrating epithelial collectives. This occurs if and only if cells retain strong E-cadherin-mediated cell-cell adhesion contacts, which represents a hallmark of epithelial cells. Although cells can effectively withstand both compressive and tensile stress, shear stress generated in a physiological process such as collective cell migration has the potential to: (i) rupture the adhesion contacts between cells and between cells and the extracellular matrix, (ii) lead a partial disintegration of the lipid bilayer and softening of the cytoskeleton, (iii) induce the inflammation of cells, and (iv) cause changes in gene expression. The main goal of this theoretical consideration has been to indicate the reasons for intensive accumulation of shear stress in anisotropic, directional migration of epithelial monolayers on substrate matrices and to discuss possible strategies by which cells minimise the shear stress, while pointing out the cost of these strategies. Our primary findings were derived from an analysis of the inhomogeneous dynamics associated with the wetting and de-wetting of epithelial monolayers, highlighting the physical characteristics of viscoelasticity resulting from collective cell migration. In light of the observation that both physical parameters, including cell mechanical stress and corresponding strain as well as epithelial spreading factor, illustrate the viscoelastic characteristics of cell rearrangement, it can be concluded that these parameters are associated with the degree of anisotropy. The degree of anisotropy can be associated with the comparative proportions of aligned cells oriented in the direction of migration as well as in the perpendicular direction. These results can be summarized as follows:

- In addition to shear strain, normal strain components also contribute to the generation of shear stress in anisotropic cell movement. This stress is accumulated within adhesion complexes and the cell cytoskeletons.
- If cells maintain their directional migration and exhibit strong cell-cell adhesion, they can reduce their exposure to shear stress by aligning with the direction of maximum principal stress. Nevertheless, inhomogeneous wetting and de-wetting can perturb cell alignment and force cells into rapid changes in their direction of migration, causing a local increase in shear stress.
- The response of cells to minimise shear stress accumulation can occur within either of two scenarios. One is to retain their epithelial phenotype but decrease the degree of anisotropy, while the other is associated with losing their epithelial phenotype by maintaining anisotropic cell migration. For the first scenario, an interplay between the shear and compressive stress components is needed. While compressive stress stimulates cell-cell interactions, inducing contact inhibition of locomotion, shear stress causes rotation of cells and additionally perturbs cell alignment toward random, isotropic, cell migration. The prerequisite of cells to retain their active contractile state and strong cell-cell adhesion contacts is that the time between two cell collisions is longer than that required for cell re-polarisation.
- The alternative solution for epithelial cells is to change their phenotype by undergoing the epithelial-to-mesenchymal cell state transition and maintaining anisotropic cell migration. This process is triggered by low shear stress, below 1 Pa, and the influence of compressive stress is not needed. Weakening of the cell-cell adhesion contacts characteristic of mesenchymal cells results in intensive energy dissipation. It takes place over minutes, leading to a decrease in cell shear stress.

Looking forward, several important questions remain open. A key challenge is to determine the precise thresholds of shear and compressive stress that govern whether epithelial cells preserve their collective, cohesive phenotype or undergo the epithelial-to-mesenchymal transition. Future work should aim to couple viscoelastic modelling with experimental approaches such as live imaging, traction force microscopy, and molecular markers of cell state to establish quantitative links between stress anisotropy and phenotype switching. In addition, investigating how substrate stiffness, extracellular matrix organization, and biochemical signalling pathways modulate stress accumulation may provide new insights into how collective migration is regulated in development, wound repair, and cancer invasion. Addressing these questions will not only deepen the fundamental understanding of epithelial mechanics but may also inspire novel strategies for tissue engineering and therapeutic interventions.

## Data Availability

Not applicable.

## Glossary of terms

**Anisotropy**: The property of a system where its physical characteristics vary depending on the direction in which they are measured. This means that a system can exhibit different properties, like strength or stiffness, along different axes.
**Cell jamming**: refers to the transition from an active, contractile state to a passive, non-contractile state driven by the accumulation of compressive stress, which increases cell packing density. This transition has a profound impact on the viscoelastic properties and surface characteristics of multicellular systems. The anisotropic-to-isotropic transition occurs prior to cell jamming, while epithelial cells retain their phenotype.
**Epithelial-to-mesenchymal transition (EMT)**: is a biological process in which epithelial cells lose their cell–cell adhesion and polarity, acquire a more migratory and invasive mesenchymal phenotype, and remodel their cytoskeleton, enabling enhanced motility. Mesenchymal cells maintain partially anisotropic migration.
**Live cell extrusion**: is the process by which a viable cell is actively removed from an epithelial layer, typically to maintain tissue homeostasis. A transition from anisotropic to isotropic behavior occurs prior to live cell extrusion.
**Mechanical stress**: A physical quantity that describes the magnitude and direction of the force per unit area that causes a system deformation.
**Multicellular domain**: a group of neighboring cells characterized by homogeneous cell tractions and the strength of cell-cell and cell-matrix adhesion contacts. Cell packing density, velocity and mechanical stress are homogeneously distributed within a domain. The domain exists over some period of time and then loses its identity due to interactions with neighboring domains.
**Normal stress**: a stress along the direction normal to the surface. If positive (in the outward direction), the stress is tensional. If negative (in the opposite direction), the stress is compressive.
**Residual stress**: stress that remains in a system in the absence of external forces. It can be either normal or shear, dissipative (viscous) or elastic.
**Strain**: The deformation of a system in response to mechanical stress.
**Shear strain**: deformation of a system in response to mechanical stress applied tangentially.
**Shear stress**: stress that acts coplanar with the cross section of a system.
**Viscoelasticity**: The time-dependent response of a cellular system under externally or internally applied forces. It includes energy storage and dissipation during cell rearrangement. The mechanism of energy storage and dissipation is closely connected with the stress and strain relaxation phenomena and can be described in the form of a proper constitutive model.
**Viscoelasticity of epithelial monolayers**: Epithelial monolayers behave as linear viscoelastic solids. This behaviour, confirmed in various experimental systems, results primarily in the ability of epithelial cells to establish strong E-cadherin-mediated cell-cell adhesion contacts.
